# Influence of Environmental Conditions Associated with Low and High Altitudes on Economic and Quality Characteristics of Fruit Ripening of *Camellia chekiangoleosa* Hu

**DOI:** 10.3390/foods14132266

**Published:** 2025-06-26

**Authors:** Teng Wei, Shengyue Zhong, Bin Huang, Kang Zha, Jing Li, Qiang Wen

**Affiliations:** 1 Jiangxi Provincial Key Laboratory of Oil-Tea Camellia Resource Cultivation and Utilization, Jiangxi Academy of Forestry, Nanchang 330032, China; weitengliangshancu@outlook.com (T.W.); huangbin007@yeah.net (B.H.); 18715605056@163.com (K.Z.); 2 State Key Laboratory of Food Science and Resources, Nanchang University, Nanchang 330047, China; 3 College of Food Science and Engineering, Jiangxi Agricultural University, Nanchang 330045, China

**Keywords:** *Camellia chekiangoleosa* Hu., altitude, fruit shell, seed oil, oil yield

## Abstract

*Camellia chekiangoleosa* Hu. (*C. chekiangoleosa*) is a typical high-altitude oil-tea Camellia species. Due to altitude being an important factor affecting crop growth and quality, the influence of environmental conditions associated with low (60 m) and high (600 m) altitudes on the economic and quality characteristics of fruit ripening was assessed in this study. Our investigations showed that altitude has no influence on the growth pattern of *C. chekiangoleosa* fruit shells and seed oils, and the differences in samples between different altitudes gradually decreased with the ripening of *C. chekiangoleosa*. Nevertheless, mature *C. chekiangoleosa* fruit shells and seed oils from low and high altitudes showed some differences. Specifically, the fruit shells of *C. chekiangoleosa* cultivated in low-altitude areas contained more soluble sugar, protein, total polyphenols, total flavonoids, and tea saponin. Meanwhile, low-altitude cultivation elevated the abundance of α-tocopherol, β-sitosterol, β-amyrinol, flavonoids, and polyphenols in mature seed oils but decreased the oil yield. Moreover, few effects of altitude on fatty acid composition were observed in mature seed oils. Cluster and receiver operating characteristic (ROC) analysis indicated that the influence of altitude on the quality of mature seed oils was strongly associated with oil yield and α-tocopherol. Taken together, the present study suggests that when cultivating *C. chekiangoleosa* in low-altitude regions, more energy should be devoted to improving oil yield. The results of the fruiting process and quality trait variation in *C. chekiangoleosa* during the low-altitude introduction process can provide an important theoretical basis for the introduction and cultivation of this oil-tea species.

## 1. Introduction

*Camellia chekiangoleosa* Hu. (*C. chekiangoleosa*), of which the planting area and output of camellia oil both rank fourth among the main varieties of oil-tea camellias in China, is endemic to mountainous areas in southern China and its adjoining coverage areas. Generally, *C. chekiangoleosa* is used in edible oil, pharmaceuticals, gardening, and other industries [1]. There have been increasing reports on the nutrient value of *C. chekiangoleosa* seed oil, which is higher than that of *Camellia oleifera* (*C. oleifera*) seed oil [1]. *C. chekiangoleosa* oil provides approximately 60% oil, which is more than that of *C. oleifera* (40%). Moreover, *C. chekiangoleosa* oil is characterized by high oleic acid, comprising more than 70% of the total oil content [1,2]. Interestingly, oils with high oleic acid have been reported to be beneficial, such as reducing cholesterol and triglyceride levels in the blood [3]. Moreover, oleic acid possesses high oxidative stability, making this oil have wide applications, such as advanced natural skincare cosmetics [3]. Furthermore, *C. chekiangoleosa* oil is rich in natural antioxidants, including squalene, phytosterol (mainly that of β-sitosterol), polyphenols, fat-soluble vitamins (Vitamins A, B, and E), and tea saponin. These components possess different biological activities in lowering triglycerides (TAGs) and cholesterol, which prevents arteriosclerosis, heart disease, hypertension [4], and other diseases [5,6].

In addition, camellia fruit shells are another important biomass resource, but camellia fruit shells are generally ignored and wasted in the camellia industry. Reportedly, camellia fruit consists of 50–60% of the fruit shell. However, approximately 3.0 × 10^6^ tons of fruit shells are discarded or burned every year, resulting in enormous waste of carbon sources and serious environmental pollution [7]. Camellia fruit shells contain not only hemicellulose, cellulose, and lignin, which can be utilized to produce platform compounds, polymer materials, and liquid fuels, but also 8% tea saponin and a certain amount of polyphenols [8]. Nowadays, the extraction and processing of active ingredients such as saponin and polyphenols has been the main field in the industrialization of fruit shells [9,10]. Tea saponin has good characteristics, such as strong foaming, emulsifying, dispersing, and wetting performances, and anti-cancer, anti-inflammatory, antibacterial, and other biological activities; therefore, it has been widely used in food, medicine, pesticides, and other fields [11]. Polyphenols are useful for anti-obesity treatment, scavenging free radicals, lowering blood pressure and blood lipids, and anti-allergy, antioxidant, and anti-inflammatory treatments, etc., and are widely used in medicine, food, cosmetics, and other industries [12,13]. Compared with *C. oleifera*, *C. chekiangoleosa* has a thicker fruit shell, accounting for 70–80% of the mass of the total fruit. Therefore, strengthening the development of high-value-added, comprehensive utilization of the fruit shells plays a key role in promoting the healthy and sustainable development of the *C. chekiangoleosa* industry.

In general, *C. chekiangoleosa* is naturally planted in the mountains at an elevation of 600–1400 m at the junction of five provinces (southern Zhejiang, southern Anhui, northeastern Jiangxi, southern Hunan, and northern Fujian) in China [14]. A complex growth environment greatly hinders the development and breeding of *C. chekiangoleosa*. For example, Fu et al. [15] examined the relationship between phenotypic traits and soil properties across 198 accessions from 13 populations. They found that *C. chekiangoleosa* seed oil yield was negative to total nitrogen, hydrolyzed nitrogen, total phosphorus, available phosphorus, and available potassium. Moreover, Ruan explored the effects of compound fertilizer combined with bio-organic fertilizer on the growth of *C. chekiangoleosa*. It was found that excessive fertilizer application tends to cause negative effects on tree height and crown width of *C. chekiangoleosa* [16]. However, the standard for soil fertility has not yet been reported; therefore, more work is needed to explore the influence of complex environmental conditions on *C. chekiangoleosa* growth. Nowadays, more studies have increasingly focused on altitudinal effects in *C. chekiangoleosa* growth to inform its introduction from high- to low-altitude regions. For instance, Xue and Li [17] successfully introduced *C. chekiangoleosa* and *Camellia reticulata* from western Yunnan (2000 m) to Shannxi hilly terrain (700 m) and evaluated their growth status. In fact, altitude influences not only camellia tree growth parameters but also fruit bearing and fruit quality [18,19,20]. Liu, Li, Wen, Huang, Rong, Liu, and Deng [20] systematically analyzed the influences of altitude on *C. oleifera* seed oil nutrition components and reported a negative relationship between altitude and oil yield and a positive correlation between elevation and polyunsaturated fatty acids, α-tocopherol, and tea saponin. Nevertheless, current evidence on how altitude affects camellia fruit and seed oil is still limited and most concentrated on *C. oleifera*. The related knowledge on *C. chekiangoleosa* is very limited, but such studies are important for guiding the low-altitude cultivation of *C. chekiangoleosa* and the efficient utilization of seed oils and camellia fruit shells.

In this study, the specific goals are to clarify whether altitude influences the growth pattern of *C. chekiangoleosa* fruit shells and seed oils; to study the effects of altitude on the polyphenol, flavonoid, tea saponin, fatty acid, and lipid concomitant composition of mature *C. chekiangoleosa* fruit shells and seed oils; and further, to clarify how altitude affects the quality of mature seed oils based on multivariate statistical analysis. The information provided by this study could be useful for guiding the low-altitude cultivation of *C. chekiangoleosa* and the efficient utilization of *C. chekiangoleosa* fruit shells and seed oils.

## 2. Materials and Method

### 2.1. Sample Collection

**Figure 1:**
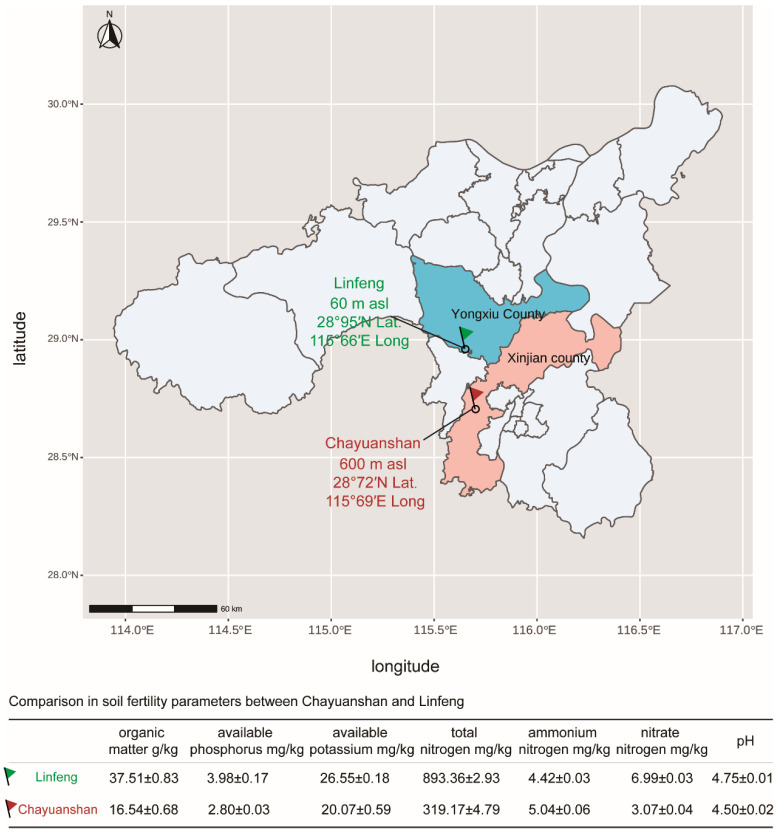
Cultivation of *C. chekiangoleosa* and soil fertility parameters.

We collected four clones of different genotypes (LK29, LK15, KH13, and KH03) of *C. chekiangoleosa* during the ripening stage at five harvesting dates spaced at about 14-day intervals, starting on 5 July 2022 and ending on 2 September 2022. We harvested the high-altitude cultivated *C. chekiangoleosa* from the Chayuanshan Forestry Farm of Nanchang city, Jiangxi Province (600 m asl, 28°72′ N Lat. and 115°69′ E Long), and low-altitude cultivated *C. chekiangoleosa* from the Linfeng Forestry Farm of Jiujiang city, Jiangxi Province (60 m asl, 28°95′ N Lat. and 115°66′ E Long) (Figure 1). The *C. chekiangoleosa* trees from both regions have reached the peak fruiting stage, and the soil fertility factors are shown in Figure 1. Compared with Chayuanshan with its 600 m elevation, the overall soil fertility of Linfeng at 60 m elevation is higher, which is reflected in higher organic matter, available phosphorus, available potassium, total nitrogen, and nitrate nitrogen contents. For every testing point, we selected ten individuals randomly from each clone and picked fifteen fruits randomly from every plant. The fresh fruits from the higher altitude (LK29H, LK15H, KH13H, and KH03H) and the lower altitude (LK29L, LK15L, KH13L, and KH03L) were sealed in plastic bags and taken back to the laboratory. Subsequently, we separated the fruits from the shells and further removed the fruit husks using a hammer to obtain the seed kernels. The seed kernels and fruit shells were dried and ground into powder with a blender and then stored at 4 °C until the analysis process.

### 2.2. Chemicals and Standards

Squalene, β-amyrinol, glucose, α-tocopherol, gallic acid, catechins, 5α-cholesterol, and β-sitosterol standards were purchased from Yuan ye Bio-Technology Co., Ltd. (Shanghai, China). Oxalic acid and methyl acetate were provided by Aladdin Industrial Corporation (Shanghai, China). The standard fatty acid methyl ester (FAME mixture #463, >99%) was bought from Nu-Chek Prep Inc. (Elysian, MN, USA). Both n-hexane and methanol were of chromatographic reagent grade and obtained from Honeywell International. Meanwhile, other solvents were of analytical reagent grade and purchased from Xilong Scientific Co., Ltd. (Shanghai, China).

### 2.3. Fruit Shell Macronutrient Analysis

#### 2.3.1. Moisture Content

Fruit shells were dried at 105 °C in an oven (DGG-9140, Senxin experiment instrument Co., LTD., Shanghai, China) until they reached constant weight, and the moisture content was determined gravimetrically.

#### 2.3.2. Oil Content

The total oil content of fruit shells was analyzed with Soxhlet equipment (SXT-06, Hongji Co., Shanghai, China). In brief, petroleum ether was used to extract the fat of dried seed kernel powders and fruit shell powders at 50 °C for 8 h. Next, the extraction bottle was dried to a constant weight at 105 °C, and the increased weight was documented as the mass of total oil.

#### 2.3.3. Soluble Sugar and Starch Content

Wei et al. [21] described the analytical method for evaluating soluble sugar and starch in camellia seed. Briefly, de-oiled samples obtained from the method described in Section 2.3.2 were first used to extract soluble sugar. Then, HCl was used to hydrolyze the insoluble residue to produce soluble sugar, which was further mixed with 5.5 mL of anthrone reagent. After heating for 10 min in a boiling water bath, the absorbance of the mixture was measured at 620 nm (Synergy HTX, BioTek Instruments, Inc., Winooski, VT, USA).

#### 2.3.4. Protein Content

Approximately 0.5 g of the samples was digested with 0.2 g K_2_SO_4_, 3 g CuSO_4_, and 10 mL H_2_SO_4_ (98%) using a digester equipped with a temperature control system for 5 h (K9860, Haineng Future Technology Group Co., Ltd., Shandong, China). The blank sample was prepared by digesting 2.0 mL of water at the same time. Then, both sample and blank digests were mixed with NaOH and steam-distilled, which produced free ammonia. The ammonia was collected via a solution of 2% boric acid, and nitrogen content was determined by titration with 0.1 mol/L HCl after blank correction [22].

### 2.4. Analysis of Total Polyphenol and Total Flavonoid Contents in Fruit Shell

Approximately 0.1 g of the samples was dissolved in 2 mL of a 70% methanol aqueous solution. For total polyphenol analysis, a 25 μL sample was mixed with 125 μL of Folin–Ciocalteu reagent for 10 min. Then, 125 μL of 7.5% sodium carbonate was supplemented and kept in a shaker for 0.5 h. For total flavonoid content analysis, a 25 μL sample was mixed with 125 μL of NaNO_2_ (0.066 mol/L) for 5 min, and then 15 μL of AlCl_3_ (0.75 mol/L) and 100 μL of NaOH (0.5 mol/L) were added and reacted for 15 min. The absorbance value was determined at a wavelength of 765 nm for total polyphenols and 510 nm for total flavonoids. Gallic acid and catechins were used to make standard curves for total polyphenol and total flavonoid content analysis separately. The values of total polyphenol and total flavonoid contents are presented as mg of gallic acid and catechin equivalents/g of dried weight (namely, mg GAE/g and mg CE/g), respectively.

### 2.5. Fruit Shell Tea Saponin Analysis

Approximately 0.1 g of the samples was dissolved in 100 mL of 80% ethanol, and 0.5 mL of solution was moved into 25 mL tubes and mixed with 1.5 mL of ultrapure water and 1 mL of 8% vanillin ethanol solution. The mixture was added and reacted with 5 mL of a 77% sulfuric acid solution at 60 °C for 15 min. The absorbance value was determined at the wavelength of 550 nm for tea saponin content.

### 2.6. Fresh Seed Yield and Kernel Yield

The fresh seed rate is presented as the ratio of the total mass of fresh seeds to the total mass of fresh fruit (%). Similarly, kernel yield is expressed as the ratio of dry kernel weight to dry seed weight (%).

### 2.7. Seed Oil Yield and Oil Extraction

Seed oil yield was analyzed according to the method described in Section 2.3.2. Moreover, for lipid concomitant determination, seed oil was prepared by removing petroleum ether under vacuum after 8 h of Soxhlet extraction.

### 2.8. Oil Component Determination

#### 2.8.1. α-Tocopherol Determination

The oil sample (0.1 g) was transformed in a volumetric flask and made up to 1 mL with HPLC-grade hexane. The solution was injected into an Agilent 1100 series HPLC (Shanghai, China) apparatus equipped with a Hypersil ODS2 column (4.6 mm × 150 mm, 5 μm, Elite, Dalian, China) at an injection volume of 10 μL, achieving the analysis of tocopherol content. The mobile phase for α-tocopherol separation consisted of 96% methanol and 4% water, of which the flow rate was 0.8 mL/min. The column temperature was 25 °C. The absorbance value was determined at the wavelength of 292 nm for α-tocopherol content [21].

#### 2.8.2. Analysis of β-Sitosterol, β-Amyrinol, and Squalene

Zhong et al. [23] described an approach to saponification of oil samples. In brief, the oil samples of 0.1 g were thoroughly mixed with 2 mL of 2 mol/L KOH–ethanol in glass tubes. Then, 100 μL of 1 mg/mL 5α-cholesterol was added into the tubes as an internal standard, and the tubes were kept at 80 °C in a water bath for 30 min. The mixtures were mixed with 3 mL of water and further extracted with 5 mL of hexane three times. The combined hexane layer was washed three times with 10% ethanol/water (*v*/*v*) and trimethylsilyl derivatization was then achieved at 60 °C for 1 h. Next, the sample was injected into the GC-MS (gas chromatography–mass spectrometry) instrument (Santa Clara, CA, USA) with an injection volume of 1 μL. The temperature program was as follows: the initial temperature was 200 °C maintained for 0.5 min and then increased to 300 °C at a rate of 10 °C/min and held for 20 min. The inlet temperature was 280 °C, the oven temperature was 200 °C, the carrier gas was hydrogen, the carrier gas flow rate was 1.2 mL/min, and the split ratio was 50:1. Quantitative analysis of sterols and squalene was carried out using internal and external standard methods, respectively.

#### 2.8.3. Total Polyphenol and Total Flavonoid Contents

Total polyphenol and total flavonoid contents in seed oil were analyzed according to the method described in Section 2.4.

#### 2.8.4. Fatty Acid Composition Analysis of Camellia Seed Oils

The analysis procedure for fatty acid composition was described in our previous publication [24]. Briefly, 2 mg of the oil sample was converted to FAME by reaction with 40 μL of methyl acetate and 100 μL of sodium methoxide. The FAME was separated via gas chromatography (6890N, Agilent Technologies, Santa Clara, CA, USA) equipped with a CP-Sil88 column (CP7489, 100 m × 0.25 mm × 0.2 μm, Chrompack; Agilent, Santa Clara, CA, USA), thus fatty acid composition analysis was accomplished. Also, the conditions for gas chromatography are described in the abovementioned publication.

### 2.9. Statistical Analysis

All experiments were performed in triplicate, and the data were expressed as the mean value ± standard deviation. All statistical analyses were performed using GraphPad Prism 8.0 (GraphPad Software Inc., San Diego, CA, USA). One-way ANOVA analysis with Tukey’s test was used to compare the differences between mean values. A level of probability of *p* < 0.05 was set as statistically significant. Principal component analysis (PCA) was performed using Statistical Product and Service Solutions (SPSS) 23.0 (IBM, Armonk, NY, USA). Hierarchical clustering, K-means clustering, and ROC analysis were achieved using the OmicStudio tools at https://www.omicstudio.cn/tool (accessed on 20 October 2023).

## 3. Results and Discussion

### 3.1. Effect of Altitude on Chemical Composition of C. chekiangoleosa Fruit Shell During the Ripening Process

Camellia fruit shell accounts for 50–60% of the mass of camellia fruit, and the output of camellia fruit shell is extremely huge. But so far, over 3.0 × 106 tons of fruit shell have been directly discarded or burned every year, causing quite a lot of waste of carbon sources and bringing about environmental pollution [8]. It is not clear whether altitude influences the *C. chekiangoleosa* fruit shell’s ripening process and final composition, the investigation of which contributes to the efficient utilization and sustainable development of *C. chekiangoleosa* fruit shell.

As shown in Table 1, moisture is the most important component in the *C. chekiangoleosa* fruit shell. Moisture content gradually increased from 60.35–64.59 g/100 g shell on 5 July to 75.27–77.42 g/100 g shell on 3 August, and subsequently remained constant. Due to absorbing moisture from the soil for photosynthesis during the early life stage of *C. chekiangoleosa* fruit, the moisture content of seeds is relatively high at this stage. Overall, at the early life stage of *C. chekiangoleosa*, the fruit shell’s moisture content was found to be higher in the low-altitude region than in the high-altitude region. There is limited evidence investigating the correlation between camellia fruit shell moisture content and altitude. However, a study on apricots found that fruit moisture content decreased significantly with an increase in elevation. Naryal et al. [25] attributed the decrease in moisture content to the dry climatic conditions caused by increased elevations. In addition, *C. chekiangoleosa* fruit shells contained 6.62–14.64 g/100 g shell of soluble sugar, 8.05–16.25 g/100 g shell of starch, 0.68–3.59 g/100 g shell of protein, and 0.39–2.65 g/100 g shell of oil. Specifically, *C. chekiangoleosa* fruit shells contained 8.47–10.19 g/100 g shell of soluble sugar on 5 July, which subsequently increased to 11.24–14.64 g/100 g shell on 3 August and 17 August, and finally decreased to 6.62–13.03 g/100 g shell on 2 September. The trends of starch content were opposite to those of soluble sugar; they changed from 11.62–14.68 g/100 g shell on 5 July to 8.05–10.68 g/100 g shell on 3 August to 9.51–16.25 g/100 g shell on 2 September Protein and oil in *C. chekiangoleosa* fruit shells increased from 0.77–1.42 g/100 g shell and 0.39–1.01 g/100 g shell to 2.29–3.25 g/100 g shell and 1.83–2.65 g/100 g shell, respectively, with the ripening of *C. chekiangoleosa*. The initial increase in soluble sugar content might be associated with the hydrolysis of starch, which was reflected in the decreased starch content. Subsequently, the produced soluble sugar was converted into protein and oil via the phosphoenolpyruvate pathway [26], thus resulting in a gradual increase in protein and oil content. At the later stage of the ripening process, amylase activity is reduced in the fruit shell, which has been observed in various plants, such as eggplants and strawberries [27,28], thus resulting in the accumulation of starch in *C. chekiangoleosa* fruit shells. In conclusion, altitude did not change the growth trends of macronutrients during the ripening of *C. chekiangoleosa*.

Table 1 further shows the effect of altitude on the total polyphenol, total flavonoid, and tea saponin contents of the *C. chekiangoleosa* fruit shell during the ripening process. During the ripening process of *C. chekiangoleosa*, both total polyphenols and total flavonoids were initially increased, followed by a decreased trend. This finding was in agreement with the results that Xiang [29] and Alina et al. [30] observed in *C. oleifera* and plum peels. Increased polyphenols and flavonoids at early life stages might protect the development of *C. chekiangoleosa* fruit by inhibiting the occurrence of pathogenic bacteria and clearing active oxygen [31]. Tea saponin is one of the most important components of camellia and possesses a wide industrial perspective. During the mature process of *C. chekiangoleosa*, tea saponin content exhibited an initial decrease followed by an increase, with 134.9–167.3 mg/g on 5 July decreasing to 98.6–132.6 mg/g on 19 Jul before gradually increasing to 175.8–256.4 mg/g. Notably, similar findings have been observed in *C. oleifera* shells. Ning et al. [32] found that saponin content in *C. oleifera* shells changed from 11.80 g/100 g shell to 10.02 g/100 g shell to 12.28 g/100 g shell across five different ripening stages. We also noted the fact that tea saponin levels were significantly higher in *C. chekiangoleosa* shells than in *C. oleifera* shells across the entire ripening process [32]. In fact, tea saponin reportedly enhances plant growth and improves nutrition uptake [33,34]. So, we suggest that the shorter developmental cycles of *C. chekiangoleosa* might be associated with the accumulated tea saponin; however, further investigation is needed. In summary, it was found that trends of total polyphenol, total flavonoid, and tea saponin contents were the same among all samples during the ripening. And compared to high-altitude cultivation, the total polyphenol, total flavonoid, and tea saponin contents in fruit shells of low-altitude cultivated *C. chekiangoleosa* were higher.

### 3.2. Effect of Altitude on Shell Chemical Composition of Mature C. chekiangoleosa Fruit

**Figure 2:**
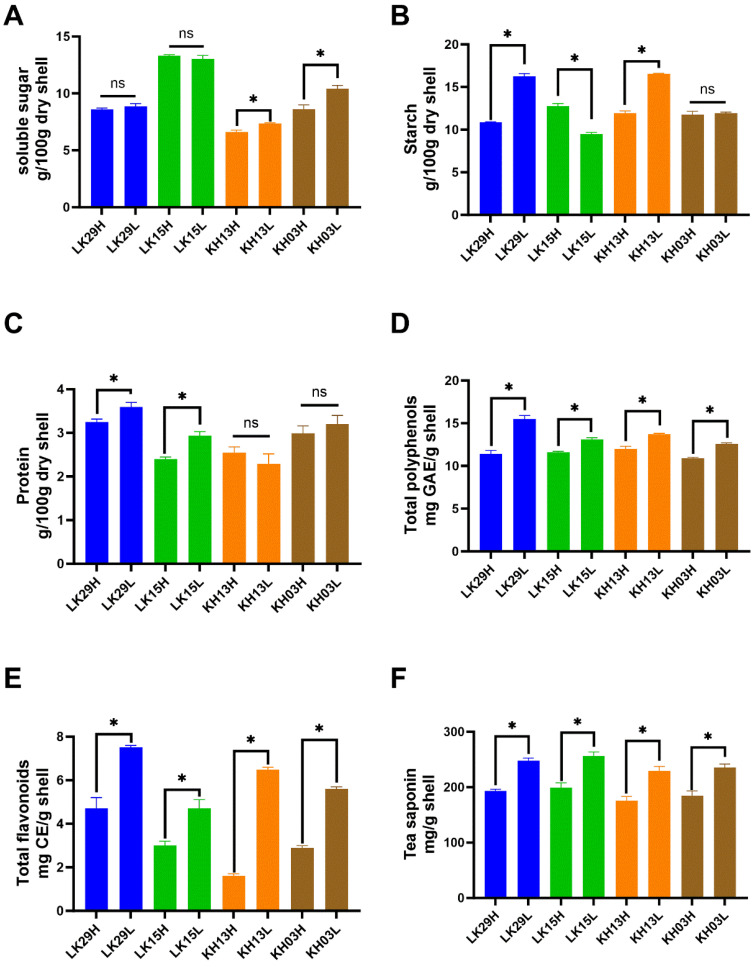
Effect of altitude on the chemical composition of mature fruit shells of *C. chekiangoleosa* (dried weight). (**A**) Soluble sugar content; (**B**) starch content; (**C**) protein content; (**D**) total polyphenols; (**E**) total flavonoids; and (**F**) tea saponin. Note: One-way ANOVA was used to compare the differences between mean values (*p* < 0.05); the figures show the results within the same asexual lineage. “*”: *p* < 0.05; “ns”: *p* > 0.05.

**Figure 3:**
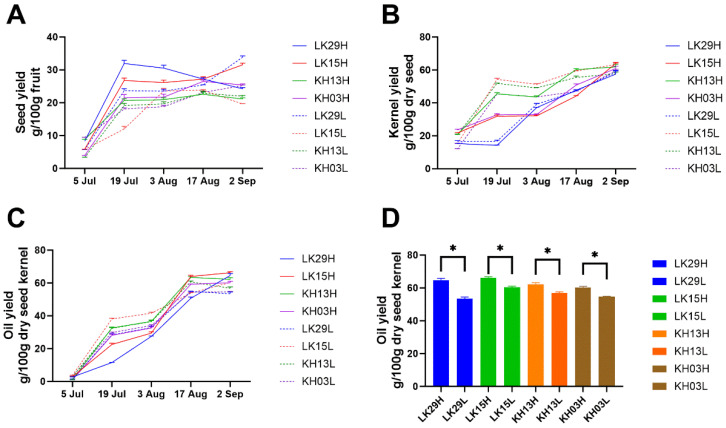
Effect of altitude on seed yield (**A**), seed kernel yield (**B**), and oil yield (**C**) during the ripening of *C. chekiangoleosa* seeds; (**D**) the effect of altitude on the oil yield of mature seed. ANOVA was used to compare the differences between mean values (*p* < 0.05); the figures show the results within the same asexual lineage. “*”: *p* < 0.05.

As shown in Figure 2A–C, the effects of altitude on mature fruit shell soluble sugar, starch, and protein contents were various among different clones. Low-altitude cultivation significantly increased the soluble sugar and protein contents in LK29, the protein content in LK15, the soluble sugar and starch contents in KH13, and the soluble sugar content in KH03 compared to high-altitude cultivation. However, decreased starch content was observed in KH13 cultivated in low-altitude regions, while altitude had no influence on soluble sugar content in LK29 and LK15, starch content in KH03, or protein content in KH13 and LKH03. Taken together, altitude had a negative effect on fruit shell soluble sugar, starch, and protein contents. One possible reason is that the high oil yield (Figure 3D) observed in mature *C. chekiangoleosa* seeds comes at the cost of consuming fruit-soluble sugars, starch, and proteins.

In contrast, the effects of altitude on mature fruit shell total polyphenol, total flavonoid, and tea saponin contents were consistent among various clones. As depicted in Figure 2D–F, altitude showed a negative influence on total polyphenol, total flavonoid, and tea saponin contents in fruit shells. It is known that solar UV radiation increases with an increase in altitude. Therefore, with greater UV radiation at higher altitudes, plants were expected to show increased contents of polyphenols, flavonoids, and tea saponin and better antioxidant properties [35,36]. Our results regarding polyphenol, flavonoid, and tea saponin contents suggest that *C. chekiangoleosa* fruit shells might experience the opposite effect since all clones cultivated in low-altitude regions contained higher contents of polyphenols and flavonoids. These findings are in agreement with those obtained by Carrillo et al. [37] and Mateus et al. [38], who reported a negative effect of altitude on polyphenol content in beans and grape skin. Complex factors might contribute to the changed polyphenol, flavonoid, and tea saponin contents. For example, a lower concentration of carbon dioxide was generally observed in higher altitude areas [18,39], which might restrict the biosynthesis of polyphenols, flavonoids, and tea saponin. In fact, soil fertility is also an important factor for polyphenolic compound metabolism. In this study, the high soil ammonium nitrogen/total nitrogen at high altitude (Chayuanshan) might contribute to the low total polyphenol and flavonoid levels in *C. chekiangoleosa* fruit shells. According to Naseri et al. [40] and Chrysargyris et al. [41], decreasing phenolic content in plant tissues (leaves and roots) due to the supply of ammonium nitrogen as the sole N source was reported in several plant species (pea, corn, purslane, etc.). In this case, ammonium may alter intracellular acidity and thus affect the biosynthesis of metabolites, including the phenylpropanoid pathway that produces a series of secondary metabolites. On the other hand, most of the precursors for phenolic compound synthesis are phosphoenolpyruvate and erythritose-4-phosphate, which are consumed by sugar metabolism and lipid metabolism during *C. chekiangoleosa* fruit ripening to generate oil and protein, etc. [42]. We observed that high-altitude cultivation generally resulted in higher oil yields (Figure 3D), which might suppress the conversion of phosphoenolpyruvate to polyphenols and flavonoids. Nonetheless, further investigations are required to conclude on this subject.

### 3.3. Effect of Altitude on the Seed Yield and Oil Yield of C. chekiangoleosa During the Ripening Process

With the development of *C. chekiangoleosa* fruit, *C. chekiangoleosa* seed is subject to dramatic change, involving a phase transition from hydrogel state to solid state. This contributes to the increased seed yield and seed kernel yield. The seed yield of *C. chekiangoleosa* (LK29, LK15, KH03, and KH13) was approximately 19.61–31.54 g/100 g fruit at its mature stage (Figure 3A) when the seed kernel yield was 57.73–63.99 g/100 g seed (Figure 3B). Interestingly, higher altitude contributed to a higher seed yield (Figure 3A) while resulting in a lower seed kernel yield (Figure 3B), particularly at the early stage of *C. chekiangoleosa*. This finding suggests that when the seed kernels of low-altitude *C. chekiangoleosa* develop rapidly in the early stages, higher-altitude *C. chekiangoleosa* seeds form more endocarp. In fact, the formation of endocarp might protect the subsequent development of the seed kernel from low temperatures induced by increased elevation.

As one of the most important oil crops, oil yield is the most important economic indicator of *C. chekiangoleosa*. We therefore investigated the effects of altitude on oil yield during the ripening of *C. chekiangoleosa* seed (Figure 3C). Oil content gradually increased from 1.01–3.82 g/100 g kernel on 5 Jul to 53.48–66.27 g/100 g kernel on 2 Sep in seeds with the development of *C. chekiangoleosa*. The oil yield of mature seed is consistent with the results of the study by He et al. [43], which reported that the oil yield of *C. chekiangoleosa* seed from six producing areas was 58.46% on average. In addition, altitude indeed altered the accumulation pattern of *C. chekiangoleosa* seed oil. In general, low altitude benefited the oil accumulation at the early stage of *C. chekiangoleosa* seeds, which was consistent with the higher seed kernel yield observed at low elevation. Both of these indicated that low-altitude cultivation benefited the early development of the *C. chekiangoleosa* seed kernel. Nevertheless, *C. chekiangoleosa* seeds cultivated in high-altitude areas generally showed a higher oil yield at their mature stage (Figure 3D). In fact, He, Wu, Dong, Wen, Li, Li, Zhu, Xu, and Xu [43] and Ding et al. [44] reported the same results in camellia and peony: increasing altitude will increase the oil yield in mature seeds. For *C. chekiangoleosa* seeds cultivated in high-altitude regions, a higher oil yield was attributed to the rapid accumulation of seed oil from 3 August to 2 September. In fact, during this stage, both increased temperatures and the previously formed endocarp provide an environment for kernel development and lipid accumulation. Meanwhile, an increased photosynthetic rate with an increase in elevation greatly promoted kernel lipid biosynthesis. In fact, as Xu [45] reported in spring rape, the photosynthetic rate was positively associated with altitude. Combined, these factors contributed to rapid fat accumulation in seed from 3 August to 2 September in high-altitude regions, thereby resulting in a higher fat yield at the *C. chekiangoleosa* seed mature stage.

### 3.4. Effect of Altitude on Lipid Concomitant and Fatty Acid Composition of C. chekiangoleosa Seed Oils During the Ripening Process

Table 2 shows the effect of altitude on the lipid concomitant of *C. chekiangoleosa* seed oils during the ripening process. Total polyphenol, total flavonoid, and α-tocopherol contents increased from 0.53–4.86 mg GAE/g, 0.53–1.48 mg CE/g, and 82.17–113.57 mg/g on 5 July to 2.15–9.81 mg GAE/g, 0.65–2.00 mg CE/g, and 427.75–573.16 mg/g on 3 August, respectively. Then, they, respectively, decreased to 0.33–1.90 mg GAE/g, 0.15–0.44 mg CE/g, and 203.99–298.91 mg/g on 2 September. Squalene, β-amyrinol, and β-sitosterol contents gradually decreased from 2.03–2.82 mg/g, 2.52–3.98 mg/g, and 3.32–5.70 mg/g on 5 July to 0.53–0.61 mg/g, 0.80–1.25 mg/g, and 0.64–1.15 mg/g on 2 September, respectively.

The changes in α-tocopherol, squalene, β-amyrinol, and β-sitosterol contents within the mature process of *C. chekiangoleosa* seed oils were consistent with our previous report [21]. For total polyphenols and total flavonoids, this tendency was in agreement with the results in olive oil obtained by different authors [42,46]. They reported an increase in total polyphenols and total flavonoids in olive oil during fruit ripening, followed by a considerable decrease in fully mature oil. Thumbnail images show the trends in average value from high- and low-altitude regions. *C. chekiangoleosa* seed oils from high-altitude regions contained more polyphenols, α-tocopherol, and β-amyrinol, and less squalene and flavonoids than those cultivated at low altitude. The differences between seed oils from high- and low-altitude regions were mainly observed on 3 August and 17 August and gradually decreased as the mature process of seed oils continued. As previously stated, from 3 August to 17 August, an increased photosynthetic rate with an increase in elevation promoted the conversion of phosphoenolpyruvate not only to lipids but also to polyphenolic compounds.

**Figure 4:**
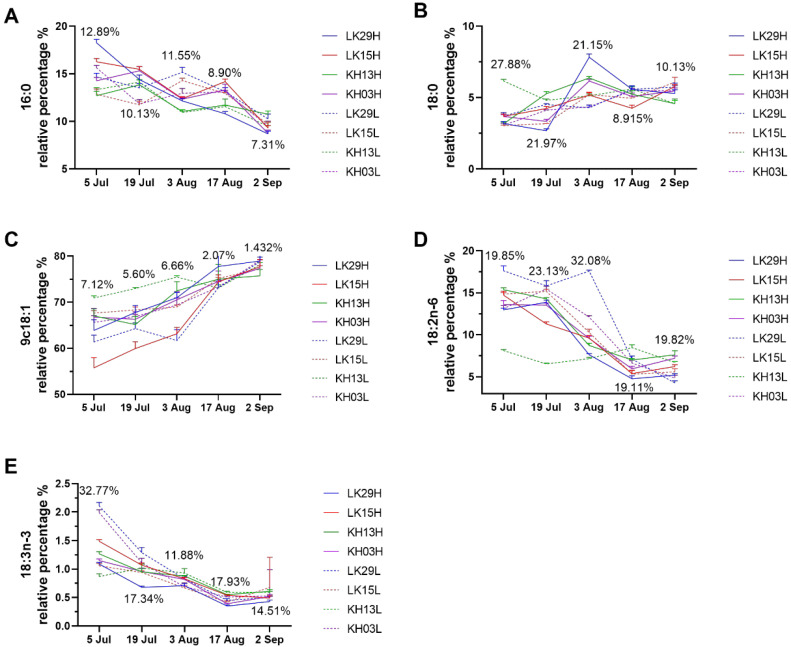
Effect of altitude on the major fatty acid composition of seed oils during the ripening of *C. chekiangoleosa*. (**A**) 16:0; (**B**) 18:0; (**C**) 9 c18:1; (**D**) 18:2 n-6; and (**E**) 18:3 n-3.

Eleven kinds of fatty acids were identified in *C. chekiangoleosa* seed oils (Tables S1–S4). In all samples studied, the main fatty acids identified were palmitic acid (16:0), stearic acid (18:0), oleic acid (9 c18:1), and linoleic acid (18:2 n-6), of which 9 c18:1 is the most important fatty acid in mature *C. chekiangoleosa* seed oils, accounting for 75.74–79.09% of total oil. Figure 4 shows the effect of altitude on the major fatty acids of *C. chekiangoleosa* seed oils during the ripening process. During the ripening process of *C. chekiangoleosa* seed oils, the effects of altitude on fatty acid levels varied among different clones. Nevertheless, altitude has no significant influence on the evolution patterns of 16:0, 18:0, 9 c18:1, 18:2 n-6, and 18:3 n-3 of *C. chekiangoleosa* seed oils. The levels of 16:0, 18:2 n-6, and 18:3 n-3 were decreased, while 18:0 and 9 c18:1 were increased with the extension of harvesting time, which was consistent with our previous report [21]. The change in fatty acids was possibly associated with the increased KAS and decreased FAD 2/6 expressions during camellia seed development [47,48], which finally contributed to the accumulation of 9 c18:1. In addition, it was found that the coefficient of variation (CV) value of fatty acids among all samples gradually decreased with the development of *C. chekiangoleosa* seed oils, indicating that the difference in fatty acid composition between *C. chekiangoleosa* seed oils cultivated at high and low altitudes declines during the ripening.

Taken together, the differences in lipid concomitant and fatty acid composition between high- and low-altitude cultivated *C. chekiangoleosa* seed oils gradually decreased with the extension of harvesting time.

### 3.5. Effect of Altitude on Seed Oil’s Chemical Composition of Mature C. chekiangoleosa Seed Oil

**Figure 5:**
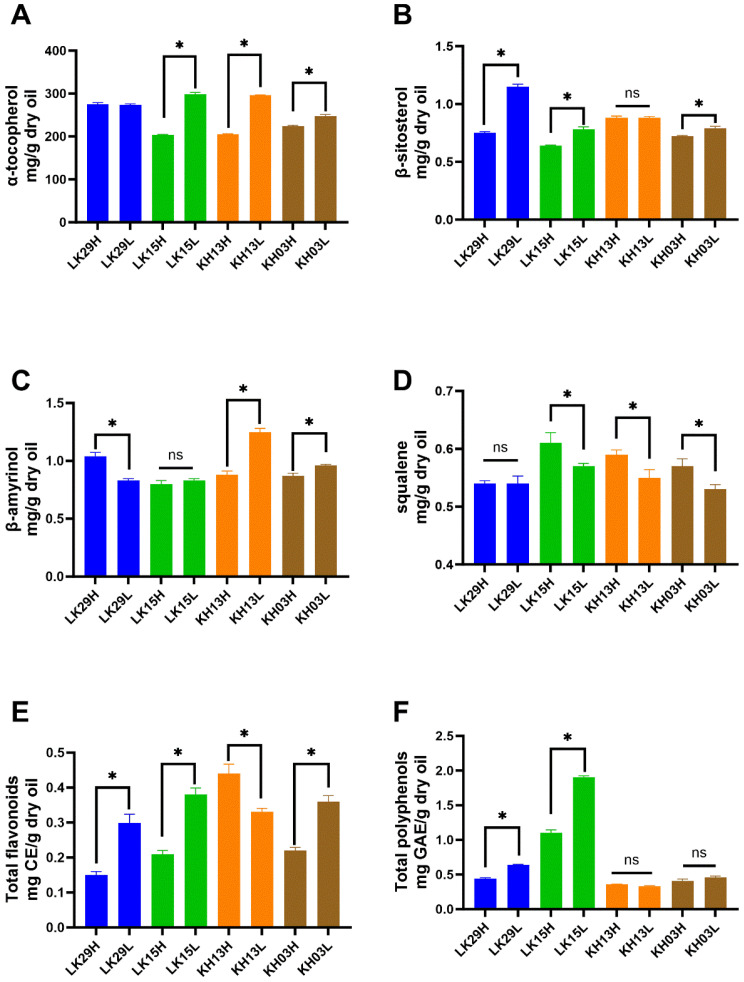
Effect of altitude on lipid concomitant composition of mature seed oils of *C. chekiangoleosa*. (**A**) α-tocopherol; (**B**) β-sitosterol; (**C**) β-amyrinol; (**D**) squalene; (**E**) total flavonoids; and (**F**) total polyphenols. Note: ANOVA was used to compare the differences between mean values (*p* < 0.05); the figures show the results within the same asexual lineage. “*”: *p* < 0.05; “ns”: *p* > 0.05.

Figure 5 shows the lipid concomitant composition of mature *C. chekiangoleosa* seed oils. Altitude influences the lipid concomitant content when seed oils matured on 2 September. Low-altitude cultivation significantly increased α-tocopherol content in LK15, KH13, and KH03; β-sitosterol content in LK29, LK15, and KH03; β-amyrinol content in KH13 and KH03; flavonoid content in LK29, LK15, and KH03; and polyphenolic content in LK29 and LK15 compared to high-altitude cultivation. In addition, compared to high-altitude cultivation, low-altitude cultivation significantly decreased β-amyrinol content in LK29, flavonoid content in KH13, and squalene content in LK15, KH13, and KH03, and had no effect on β-amyrinol content in KH13, squalene content in LK29, and polyphenolic content in KH13 and KH03. Taken together, an inclination was observed in this study that altitude showed a positive relation to squalene content and a negative association with α-tocopherol, β-sitosterol, β-amyrinol, flavonoid, and polyphenolic contents. Zhang et al. [49] also reported that phenolic compounds in olive oil were negatively correlated with altitude. This finding might be due to the temperature difference at different altitudes. The altitude of all the sample plots ranged from 900 m to 1400 m. In general, the average temperature dropped 0.5 °C for every 100 m of elevation, and the temperature difference between day and night decreased with elevation. The activities of olive cell enzymes (phenylalanine ammonialyas, PAL, etc.) are responsible for the synthesis of phenolic compounds and are influenced by temperature [50], and thus may be inhibited by the decrease in temperature. On the other hand, at the later stage of maturity, more phosphoenolpyruvate was metabolized to lipids, thereby decreasing the biosynthesis of phenolic compounds. Similar to the finding in fruit shell, the high soil ammonium nitrogen/total nitrogen in high altitude (Chayuanshan) might also contribute to the low total polyphenol and flavonoid levels in *C. chekiangoleosa* seed oils. In the plant, phytosterol was generated from squalene, which was catalyzed by squalene synthase (SQS), cycloartenol synthase (CAS1), sterol-methyl transferase (SMT), and C-4 demethylase [51]. The decrease in these enzymes caused by decreasing temperature inhibited the conversion of squalene to β-sitosterol, thus contributing to the accumulation of squalene in high-altitude cultivated *C. chekiangoleosa* seed oils. Moreover, Akbaba et al. [52] also observed that increasing altitude decreases β-sitosterol levels in the Şilfoni grape. High α-tocopherol levels observed in low-altitude cultivated *C. chekiangoleosa* seed oils might be attributed to the high organic matter content in the soil. According to Liu [53], *C. oleifera* seed oil α-tocopherol content is significantly positively associated with soil organic matter. Overall, the present study demonstrated that *C. chekiangoleosa* seed oils cultivated in low-altitude regions showed strong application prospects since their higher concentrations of natural antioxidants were useful in lowering triglycerides (TAGs) and cholesterol, thus preventing hypertension [4], heart disease, arteriosclerosis, and other diseases [5,6].

Table 3 shows the fatty acid composition of the mature *C. chekiangoleosa* seed oils. Compared to high-altitude cultivation, low-altitude cultivation significantly increased the levels of 16:0 from 8.7 ± 0.29% to 10.27 ± 0.51% in LK29 and significantly decreased 16:0 concentrations from 10.76 ± 0.34% to 9.49 ± 0.43% in LK13, but had no effect on 16:0 levels in LK15 and KH03. The concentration of 18:2 n-6 in *C. chekiangoleosa* seed oil from low-altitude regions (4.27–6.71%) was lower than that from high-altitude areas (5.2–7.63%), and significant differences were observed in LK29, KH13, and KH03. This finding differs from previous reports on 18:2 n-6 in camellia and olive oil [43,49,54]. We suggest that complex factors such as genotypes and soil conditions contribute to this difference. For example, Erel et al. [55] reported that the increasing level of ammonium nitrogen could increase the desaturation of 18:1 n-9 c. As a result, the content of 18:2 n-6 is increased in Chayuanshan at a higher altitude. However, there was no association between 9 c18:1, 18:0, and 18:3 n-3 contents and altitude, which is in accordance with the findings of He, Wu, Dong, Wen, Li, Li, Zhu, Xu, and Xu [43] and Liu, Ni, Qin, W, Fang, and Wang [19]. They also reported a weak relationship between 9 c18:1 and elevation in *C. chekiangoleosa* and *C. oleifera*, respectively. This suggests that, as the vital oleic acid source, altitude did not affect the commercial and processing value of mature *C. chekiangoleosa* seed oils. Collectively, altitude limitedly influenced the fatty acid composition of mature *C. chekiangoleosa* seed oils, which was in agreement with He et al., who analyzed the correlation between *C. chekiangoleosa* seed oil characteristic indexes and the geo-ecological factors of six production areas, and found that oil fatty acid composition was not significantly affected by altitude.

### 3.6. Cluster Analysis of C. chekiangoleosa Seed Oils

**Figure 6:**
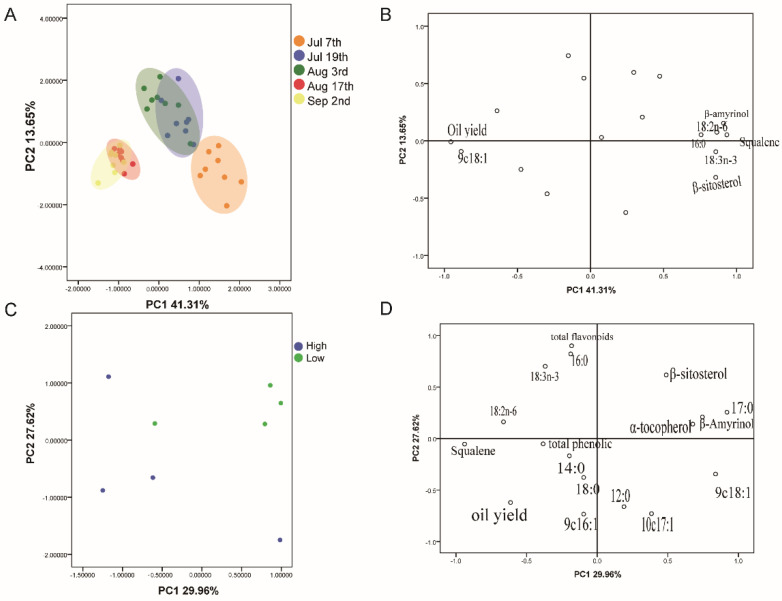
PCA analysis. (**A**) PCA analysis of seed oils collected from low- and high-altitude regions at different harvest times; (**B**) loading plot for (**A**); (**C**) PCA analysis of mature seed oils collected from low- and high-altitude regions; and (**D**) loading plot for (**C**).

PCA is used to reduce the initial variables to a small part of the principal components in order to obtain an overview of sample variations and identify patterns of behavior. Samples harvested from various harvesting times and different altitudes were used for statistical assessment; the variables comprised fatty acids, oil yield, and lipid concomitants. The loading plot of the first two components for *C. chekiangoleosa* seed oil samples is presented in Figure 6A. We note that the first two component scores take into account 54.96% (component 1 = 41.31%, and component 2 = 13.65%) of the total variation. The samples from various harvesting times are distributed along the horizontal axis from right to left. Interestingly, component 1 exhibits positive loading mainly with β-sitosterol, squalene, β-amyrinol, 16:0, 18:2 n-6, and 18:3 n-3, and negative loading with oil yield and 9 c18:1 (Figure 6B). This finding showed that oil yield and 9 c18:1 contents increased and β-sitosterol, squalene, β-amyrinol, 16:0, 18:2 n-6, and 18:3 n-3 contents decreased with the development of *C. chekiangoleosa* seed oil. Another finding is that samples gradually accumulated with the extension of harvesting time, which was significantly observed after 17 August. This suggests that the effects of altitude on seed oils would decrease with the ripening of *C. chekiangoleosa*.

**Figure 7:**
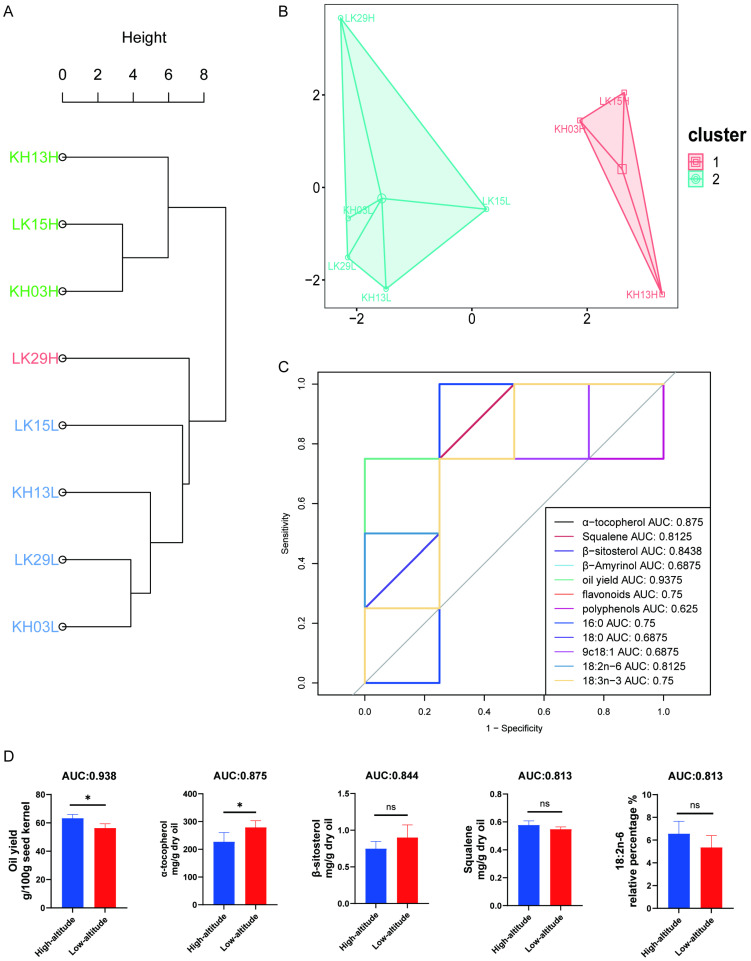
Difference analysis of mature seed oils collected from low- and high-altitude regions; (**A**) hierarchical clustering; (**B**) K-means clustering; (**C**) ROC analysis; and (**D**) T-test for critical components. “*”: *p* < 0.05; “ns”: *p* > 0.05.

Further, we carried out a PCA analysis between samples from high- and low-altitude regions to investigate how altitude influences mature seed oil quality. As depicted in Figure 6C, scatters of the high-altitude region were relatively discrete and distributed in 2, 3, and 4 quadrants, while samples of the low-altitude regions accumulated well and were distributed in 1 and 2 quadrants. According to the loading plot shown in Figure 6D, it was found that altitude showed a positive correlation to oil yield, squalene, et al., and a negative association with 17:0, β-sitosterol, β-amyrinol, and α-tocopherol. Similarly, both hierarchical clustering and K-means clustering showed that samples were divided into two clusters (Figure 7A,B). KH13H, LK15H, and KH03H belonged to cluster 1, while LK29H, LK15L, KH13L, LK29L, and KH03L consisted of cluster 2. This finding suggests that altitude significantly influenced the seed oil’s quality of KH13, LK15, and KH03 rather than LK29. To determine what components were significantly changed by altitude, we conducted a ROC analysis between high- and low-altitude samples. As shown in Figure 7C and Table 4, the AUC values of oil yield, α-tocopherol, β-sitosterol, squalene, and 18:02 exceeded 0.8, indicating a strong relationship between these five components and altitude (AUC > 0.8). This was consistent with the results of the loading diagram depicted in Figure 7D. Combined with further T-test analysis, oil yield and α-tocopherol were identified as the most different components between high- and low-altitude seed oils. Of which, oil yield increased and α-tocopherol decreased with increasing altitude. Taken together, current evidence shows that seed oils of *C. chekiangoleosa* cultivated in low-altitude regions show powerful commercial and processing prospects for their higher natural antioxidant concentrations (such as α-tocopherol, polyphenols, and flavonoids). However, decreasing elevation decreased *C. chekiangoleosa* seed oil yield. Our study suggests that, for the low-altitude cultivation of *C. chekiangoleosa*, more energy should be devoted to improving its oil yield.

## 4. Conclusions

This study investigated the dynamic evolution of fruit shells and seed oils in four clones of *C. chekiangoleosa* during ripening. In summary, altitude had no effect on the growth pattern of *C. chekiangoleosa* fruit shells and seed oils. And the effects of altitude on seed oil quality decreased with the ripening of *C. chekiangoleosa*. Nevertheless, some differences between the two altitudes were indeed discovered in mature *C. chekiangoleosa* fruit shells and seed oils. Fruit shells of *C. chekiangoleosa* cultivated in low-altitude areas had higher phytochemical contents, such as flavonoids, polyphenols, and tea saponin, than those in high-altitude regions, exhibiting larger industrial and commercial prospects. Moreover, regarding the oil quality in *C. chekiangoleosa* seeds, our investigations showed that the effects of altitude on oil quality gradually decreased with the ripening of seed oil. In mature seed oils, increasing altitude increased the oil yield and squalene contents, decreased β-sitosterol, β-amyrinol, α-tocopherol, flavonoid, and polyphenol contents, and limitedly influenced fatty acid composition. According to the results of cluster and ROC analyses, the influence of altitude on the quality of mature seed oils is strongly associated with changes in oil yield and α-tocopherol.

Given the observed relationship between soil fertility and seed oil yield in previous studies, we recommend appropriately reducing soil nitrogen, phosphorus, and potassium concentrations when cultivating *C. chekiangoleosa* in low-altitude regions. If possible, artificial supplemental light can be applied, which has been shown to be significantly positive to oil yield.

## Figures and Tables

**Table 1:** Effect of altitude on the chemical composition of fruit shells during the ripening of *C. chekiangoleosa* (dried weight of shell).

	LK29		LK15		KH13		KH03	
	High Altitude	Low Altitude	High Altitude	Low Altitude	High Altitude	Low Altitude	High Altitude	Low Altitude
Moisture (g/100 g)								
5 July	60.35 ± 1.39 a−	66.37 ± 0.86 a+	62.74 ± 0.68 b+	61.86 ± 2.29 b+	64.18 ± 2.24 c+	62.73 ± 0.84 c+	62.57 ± 0.45 d+	64.59 ± 3.29 d+
19 July	69.73 ± 2.33 a+	74.36 ± 1.57 a+	66.59 ± 1.49 b+	69.68 ± 1.38 b+	70.93 ± 1.46 c+	72.04 ± 2.24 c+	70.28 ± 1.22 d+	71.95 ± 1.72 d+
3 August	75.42 ± 1.14 a+	75.9 ± 2.23 a+	75.22 ± 2.39 b+	75.27 ± 1.69 b+	76.17 ± 2.52 c+	76.92 ± 1.49 c+	77.42 ± 1.47 d+	76 ± 0.57 d+
17 August	75.28 ± 2.78 a+	77.24 ± 2.17 a+	76.14 ± 1.39 b+	75.31 ± 0.55 b+	72.69 ± 3.11 c+	76.25 ± 2.65 c+	74.18 ± 0.95 d+	72.38 ± 0.47 d+
2 September	78.57 ± 0.66 a+	74.56 ± 3.42 a+	75.86 ± 2.78 b+	75.12 ± 1.19 b+	77.86 ± 2.05 c+	78.6 ± 1.27 c+	78.17 ± 2.61 d+	78.46 ± 2.28 d+
Oil (g/100 g)								
5 July	1.01 ± 0.03 a+	0.39 ± 0.01 a−	0.45 ± 0.01 b−	0.83 ± 0.02 b+	0.73 ± 0.02 c+	0.73 ± 0.01 c+	0.46 ± 0.01 d−	0.54 ± 0.02 d+
19 July	0.86 ± 0.02 a+	0.67 ± 0.04 a−	0.74 ± 0.03 b+	0.63 ± 0.01 b−	0.56 ± 0.01 c−	0.92 ± 0.02 c+	0.79 ± 0.02 d+	0.83 ± 0.01 d+
3 August	0.94 ± 0.02 a−	1.44 ± 0.03 a+	1.01 ± 0 b−	1.73 ± 0.01 b+	0.85 ± 0.03 c−	1.53 ± 0.03 c+	1.24 ± 0.03 d−	1.64 ± 0.03 d+
17 August	1.51 ± 0.01 a−	1.72 ± 0.06 a+	1.6 ± 0.01 b−	2.03 ± 0.03 b+	1.45 ± 0.03 c−	2.17 ± 0.02 c+	1.78 ± 0.02 d−	2.33 ± 0.01 d+
2 September	1.83 ± 0.02 a−	2.03 ± 0.05 a+	2.07 ± 0.02 b−	2.26 ± 0.04 b+	1.77 ± 0.03 c−	2.4 ± 0.03 c+	2.02 ± 0.07 d−	2.65 ± 0.02 d+
Protein (g/100 g)								
5 July	1.42 ± 0.03 a+	0.83 ± 0.01 a−	1.34 ± 0.01 b+	0.92 ± 0.02 b−	1.28 ± 0.04 c+	0.77 ± 0.01 c−	0.89 ± 0.01 d+	0.93 ± 0.02 d+
19 July	0.68 ± 0.01 a+	0.73 ± 0.01 a+	1.49 ± 0.03 b+	0.86 ± 0.02 b−	0.73 ± 0.02 c−	1.03 ± 0.01 c+	1.02 ± 0.01 d+	1.12 ± 0.05 d+
3 August	1.54 ± 0.3 a+	1.96 ± 0.13 a+	1.71 ± 0.11 b+	1.89 ± 0.05 b+	1.71 ± 0.07 c−	2.36 ± 0.2 c+	1.84 ± 0.2 d+	2.21 ± 0.1 d+
17 August	3.45 ± 0.09 a+	3.21 ± 0.1 a+	2.23 ± 0.06 b−	3.17 ± 0.02 b+	2.09 ± 0.07 c+	2.35 ± 0.2 c+	2.64 ± 0.05 d−	2.92 ± 0.05 d+
2 September	3.25 ± 0.07 a+	3.59 ± 0.11 a+	2.4 ± 0.05 b−	2.94 ± 0.09 b+	2.55 ± 0.13 c+	2.29 ± 0.23 c+	2.99 ± 0.17 d+	3.2 ± 0.2 d+
Soluble sugar (g/100 g)								
5 July	9.04 ± 0.16 a+	9.48 ± 0.07 a+	8.47 ± 0.08 b−	10.19 ± 0.14 b+	9.36 ± 0.15 c−	10.33 ± 0.42 c+	9.18 ± 0.05 d+	9.36 ± 0.22 d+
19 July	10.03 ± 0.33 a−	11.94 ± 0.15 a+	10.25 ± 0.41 b+	10.27 ± 0.24 b+	10.38 ± 0.24 c+	9.86 ± 0.11 c+	12.27 ± 0.19 d+	12.76 ± 0.09 d+
3 August	11.24 ± 0.19 a−	13.16 ± 0.07 a+	13.57 ± 0.21 b−	14.64 ± 0.2 b+	12.31 ± 0.21 c+	11.48 ± 0.24 c−	12.76 ± 0.15 d−	13.45 ± 0.16 d+
17 August	10.15 ± 0.16 a−	10.82 ± 0.13 a+	13.96 ± 0.15 b−	15.14 ± 0.18 b+	10.08 ± 0.23 c+	9.57 ± 0.29 c+	11.56 ± 0.22 d−	12.46 ± 0.15 d+
2 Sep	8.6 ± 0.12 a+	8.87 ± 0.24 a+	13.33 ± 0.06 b+	13.03 ± 0.3 b+	6.62 ± 0.15 c−	7.37 ± 0.05 c+	8.62 ± 0.37 d−	10.4 ± 0.31 d+
Starch (g/100 g)								
5 July	12.48 ± 0.11 a+	11.62 ± 0.14 a−	13.29 ± 0.26 b+	13.25 ± 0.19 b+	12.86 ± 0.13 c−	14.68 ± 0.17 c+	12.29 ± 0.25 d−	13.16 ± 0.14 d+
19 July	11.68 ± 0.21 a+	10.71 ± 0.2 a−	10.26 ± 0.17 b−	12.28 ± 0.16 b+	13.38 ± 0.24 c+	13.14 ± 0.15 c+	11.67 ± 0.18 d+	10.49 ± 0.21 d−
3 August	9.08 ± 0.11 a−	10.71 ± 0.11 a+	9.74 ± 0.12 b+	8.05 ± 0.07 b−	10.39 ± 0.13 c−	11.65 ± 0.25 c+	10.71 ± 0.15 d+	10.68 ± 0.18 d+
17 August	8.37 ± 0.19 a−	11.76 ± 0.36 a+	9.59 ± 0.24 b+	8.28 ± 0.14 b−	9.13 ± 0.01 c−	12.93 ± 0.25 c+	8.77 ± 0.23 d−	10.31 ± 0.25 d+
2 September	10.86 ± 0.07 a−	16.25 ± 0.33 a+	12.75 ± 0.32 b+	9.51 ± 0.17 b−	11.93 ± 0.27 c−	16.55 ± 0.06 c+	11.75 ± 0.41 d+	11.93 ± 0.13 d+
Total polyphenols (mg GAE/g)								
5 July	18.8 ± 0.2 a−	20.3 ± 0.2 a+	23.2 ± 0.3 b−	27.5 ± 0.3 b+	19.8 ± 0.3 c+	19.5 ± 0.2 c+	21.5 ± 0.1 d−	23.6 ± 0.1 d+
19 July	22.6 ± 0.2 a+	15.8 ± 0.3 a−	18.8 ± 0.1 b−	32.5 ± 0.2 b+	31.5 ± 0.2 c+	29.5 ± 0.3 c−	24.7 ± 0.4 d−	34.7 ± 0.5 d+
3 August	10.7 ± 0.2 a−	15.9 ± 0.3 a+	9.8 ± 0.1 b−	11.9 ± 0.1 b+	10.4 ± 0.1 c−	12.5 ± 0.1 c+	10 ± 0.1 d−	11.7 ± 0.1 d+
17 August	14.1 ± 0.2 a−	22.8 ± 0 a+	14.6 ± 0 b+	14.9 ± 0.1 b+	14.4 ± 0.2 c−	16.9 ± 0.1 c+	13.6 ± 0.1 d−	14.4 ± 0.1 d+
2 September	11.4 ± 0.4 a−	15.5 ± 0 a+	11.6 ± 0.1 b−	13.1 ± 0.2 b+	12 ± 0.3 c−	13.7 ± 0.1 c+	10.9 ± 0.1 d−	12.6 ± 0.1 d+
Total flavonoids (mg CE/g)								
5 July	8.3 ± 0.2 a+	6.5 ± 0.1 a−	7.6 ± 0.1 b+	6.9 ± 0.2 b+	6.3 ± 0.1 c−	9.2 ± 0.3 c+	7.2 ± 0.2 d−	8.5 ± 0.4 d+
19 July	11.7 ± 0.2 a−	13.4 ± 0.3 a+	12.6 ± 0.2 b+	12.7 ± 0.2 b+	10.8 ± 0.3 c−	14.5 ± 0.33 c+	11.6 ± 0.3 d+	11.5 ± 0.2 d+
3 August	15.6 ± 0.3 a−	18.5 ± 0.1 a+	14.5 ± 0.1 b−	16.5 ± 0.2 b+	15 ± 0.2 c−	18 ± 0.2 c+	17.4 ± 0.2 d+	16.6 ± 0.2 d−
17 August	18.1 ± 0.5 a−	24.2 ± 0.5 a+	17 ± 0.2 b−	18 ± 0.4 b+	13.8 ± 0.3 c−	20.4 ± 0.1 c+	16 ± 0.1 d−	18.5 ± 0.1 d+
2 September	4.7 ± 0.5 a−	7.5 ± 0.1 a+	3 ± 0.2 b−	4.7 ± 0.4 b+	1.6 ± 0.1 c−	6.5 ± 0.1 c+	2.9 ± 0.1 d−	5.6 ± 0.1 d+
Tea saponin (mg/g)								
5 July	143.9 ± 0.8 a+	134.9 ± 2.6 a−	164.7 ± 1.6 b+	167.3 ± 1.1 b+	167.2 ± 0.9 c+	153.4 ± 1 c−	157.9 ± 1.8 d+	134.9 ± 2.4 d−
19 July	113.5 ± 1.9 a+	102.7 ± 1.5 a−	105.7 ± 2.4 b−	117.3 ± 2.2 b+	132.6 ± 2.2 c+	121.7 ± 1.5 c−	98.6 ± 1.4 d+	103.9 ± 2.7 d+
3 August	162.4 ± 1.9 a+	164.3 ± 3.2 a+	149.8 ± 2.9 b−	172.4 ± 4.4 b+	147.6 ± 2.8 c−	174.7 ± 1.8 c+	152.4 ± 5.3 d−	165.3 ± 3.3 d+
17 August	143.6 ± 2.8 a−	171.6 ± 4.4 a+	151.1 ± 4.3 b−	169.5 ± 2.6 b+	134.7 ± 3.2 c−	183.6 ± 2.4 c+	146.3 ± 5.3 d−	173.6 ± 5.5 d+
2 September	193.4 ± 3 a−	248 ± 4.7 a+	199.2 ± 8.8 b+	256.4 ± 7.4 b+	175.8 ± 7.6 c−	229.4 ± 8.2 c+	184.8 ± 8.5 d−	235.3 ± 6.5 d+

Note: One-way ANOVA was used to compare the differences between mean values (*p* < 0.05); the table shows the results within the same asexual lineage. a+/a− indicates that there was a significant difference in LK29 between low altitude and high altitude; b+/b− indicates that there was a significant difference in LK15 between low altitude and high altitude; c+/c− indicates that there was a significant difference in KH13 between low altitude and high altitude; and d+/d− indicates that there was significant difference in KH03 between low altitude and high altitude.

**Table 2:** Effect of altitude on the lipid concomitant composition of seed oils during the ripening of *C. chekiangoleosa* (dried weight of seed oil).

	LK29		LK15		KH13		KH03	
	High Altitude	Low Altitude	High Altitude	Low Altitude	High Altitude	Low Altitude	High Altitude	Low Altitude
Total flavonoids (mg CE/g)								
5 Julyy	0.69 ± 0.017 a−	0.96 ± 0.038 a+	0.89 ± 0.027 b+	0.53 ± 0.01 b−	1.07 ± 0.014 c−	1.31 ± 0.047 c+	1.48 ± 0.028 d−	0.61 ± 0.025 d+
19 Julyy	1.72 ± 0.04 a−	1.98 ± 0.024 a+	1.33 ± 0.043 b+	1.19 ± 0.041 b−	1.47 ± 0.013 c−	2.5 ± 0.041 c+	0.96 ± 0.026 d−	1.41 ± 0.014 d+
3 August	0.65 ± 0.034 a−	1.71 ± 0.022 a+	1.92 ± 0.022 b+	2 ± 0.032 b+	1.3 ± 0.028 c−	1.7 ± 0.044 c+	1.24 ± 0.034 d+	0.93 ± 0.021 d−
17 August	0.3 ± 0.027 a−	0.48 ± 0.02 a+	0.63 ± 0.043 b−	1.51 ± 0.029 b+	0.4 ± 0.035 c−	0.59 ± 0.01 c+	0.41 ± 0.018 d−	0.52 ± 0.01 d+
2 September	0.15 ± 0.01 a−	0.3 ± 0.024 a+	0.21 ± 0.01 b−	0.38 ± 0.019 b+	0.44 ± 0.027 c+	0.33 ± 0.01 c−	0.22 ± 0.009 d+	0.36 ± 0.017 d−
Total polyphenols (mg GAE/g)								
5 July	4.86 ± 0.049 a+	1.95 ± 0.049 a−	4.2 ± 0.02 b+	0.95 ± 0.024 b−	4.43 ± 0.034 c+	2.58 ± 0.013 c−	0.53 ± 0.017 d−	2.27 ± 0.014 d+
19 July	2.12 ± 0.031 a+	1.4 ± 0.03 a−	2.32 ± 0.03 b+	1.63 ± 0.049 b−	2.23 ± 0.014 c−	2.63 ± 0.025 c+	2.88 ± 0.019 d+	2.36 ± 0.034 d−
3 August	7.42 ± 0.013 a+	2.15 ± 0.023 a−	8.77 ± 0.047 b+	4.72 ± 0.015 b−	8.27 ± 0.044 c+	4.63 ± 0.03 c−	9.81 ± 0.034 d+	5.92 ± 0.027 d−
17 August	1.01 ± 0.012 a+	1.07 ± 0.023 a+	1.2 ± 0.041 b−	1.41 ± 0.045 b+	0.72 ± 0.021 c−	1.19 ± 0.024 c+	0.8 ± 0.022 d−	1.29 ± 0.038 d+
2 September	0.44 ± 0.014 a−	0.64 ± 0.007 a+	1.1 ± 0.045 b−	1.9 ± 0.026 b+	0.36 ± 0.002 c+	0.33 ± 0.011 c+	0.41 ± 0.024 d+	0.46 ± 0.018 d+
α-tocopherol mg/g								
5 July	103.59 ± 0.68 a−	108.73 ± 0.85 a+	82.17 ± 0.57 b−	93.67 ± 0.95 b+	113.57 ± 0.24 c+	88.26 ± 1.09 c−	93.16 ± 1.27 d−	102.38 ± 2.26 d+
19 July	593.46 ± 4.24 a+	531.46 ± 3.26 a−	644.39 ± 0.58 b+	568.63 ± 0.62 b−	617.26 ± 0.57 c+	559.47 ± 0.89 c−	601.73 ± 4.71 d+	584.19 ± 0.57 d−
3 August	539.24 ± 0.67 a+	433.18 ± 3.33 a−	573.16 ± 1.79 b+	466.72 ± 2.12 b−	427.75 ± 2.27 c−	503.29 ± 3.51 c+	469.36 ± 2.61 d+	473.28 ± 2.62 d+
17 August	436.49 ± 4.27 a+	316.13 ± 2.64 a−	542.61 ± 0.58 b+	267.39 ± 0.62 b−	309.68 ± 0.57 c+	290.39 ± 0.94 c−	409.48 ± 2.47 d+	275.29 ± 1.27 d−
2 September	275.13 ± 3.74 a+	273.96 ± 1.74 a+	203.99 ± 0.93 b−	298.91 ± 4.07 b+	205.61 ± 0.86 c−	296.52 ± 0.41 c+	224.28 ± 1.74 d−	247.39 ± 3.82 d+
β-sitosterol mg/g								
5 July	3.23 ± 0.023 a−	4.92 ± 0.019 a+	5.7 ± 0.032 b+	5.29 ± 0.032 b−	4.85 ± 0.018 c−	5.44 ± 0.024 c+	3.85 ± 0.02 d−	4.33 ± 0.019 d+
19 July	2.19 ± 0.025 a+	2.18 ± 0.019 a+	1.61 ± 0.029 b−	2.33 ± 0.023 b+	2.41 ± 0.027 c+	1.63 ± 0.02 c−	1.96 ± 0.02 d−	2.18 ± 0.029 d+
3 August	0.98 ± 0.017 a−	1.63 ± 0.026 a+	1.84 ± 0.028 b+	1.22 ± 0.018 b−	1.83 ± 0.026 c+	1.27 ± 0.016 c−	1.1 ± 0.025 d−	1.4 ± 0.033 d+
17 August	1.21 ± 0.024 a+	0.84 ± 0.012 a−	1.49 ± 0.018 b+	1.22 ± 0.025 b−	0.96 ± 0.014 c+	0.7 ± 0.011 c−	1.17 ± 0.032 d+	0.97 ± 0.01 d−
2 September	0.75 ± 0.012 a−	1.15 ± 0.022 a+	0.64 ± 0.005 b−	0.78 ± 0.023 b+	0.88 ± 0.015 c+	0.88 ± 0.013 c+	0.72 ± 0.008 d−	0.79 ± 0.018 d+
β-amyrinol mg/g								
5 July	3.77 ± 0.018 a+	2.52 ± 0.013 a−	3.18 ± 0.009 b−	3.47 ± 0.027 b+	3.56 ± 0.014 c−	3.98 ± 0.027 c+	2.71 ± 0.026 d−	3.15 ± 0.02 d+
19 July	2.9 ± 0.016 a+	2.76 ± 0.02 a−	2.56 ± 0.015 b+	2.35 ± 0.014 b−	2.67 ± 0.027 c+	2.16 ± 0.026 c−	2.28 ± 0.028 d−	2.61 ± 0.015 d+
3 August	2.61 ± 0.03 a+	2.56 ± 0.03 a+	2.7 ± 0.013 b+	1.82 ± 0.031 b−	2.48 ± 0.027 c+	1.67 ± 0.011 c−	2.03 ± 0.009 d−	2.43 ± 0.007 d+
17 August	1 ± 0.009 a+	0.99 ± 0.012 a+	1.25 ± 0.014 b+	1.21 ± 0.011 b−	1.08 ± 0.014 c+	0.74 ± 0.006 c−	1.17 ± 0.017 d+	0.86 ± 0.023 d−
2 September	1.04 ± 0.033 a+	0.96 ± 0.027 a−	0.8 ± 0.03 b+	0.83 ± 0.015 b+	0.88 ± 0.033 c−	1.25 ± 0.03 c+	0.87 ± 0.024 d−	0.96 ± 0.009 d+
Squalene mg/g								
5 July	2.56 ± 0.024 a+	3.49 ± 0.028 a−	2.03 ± 0.011 b−	2.81 ± 0.016 b+	2.57 ± 0.026 c−	2.82 ± 0.019 c+	2.11 ± 0.035 d−	2.31 ± 0.027 d+
19 July	1.54 ± 0.019 a+	1.59 ± 0.019 a+	1.39 ± 0.028 b+	1.24 ± 0.032 b−	1.45 ± 0.011 c+	1.33 ± 0.039 c−	1.86 ± 0.022 d+	1.92 ± 0.031 d+
3 August	1.19 ± 0.017 a−	1.9 ± 0.026 a+	1.92 ± 0.025 b+	1.86 ± 0.022 b+	1.13 ± 0.019 c−	1.69 ± 0.033 c+	1.44 ± 0.022 d+	1.9 ± 0.031 d−
17 August	0.53 ± 0.017 a−	0.67 ± 0.013 a+	0.47 ± 0.008 b−	0.55 ± 0.012 b+	0.45 ± 0.006 c−	0.49 ± 0.023 c+	0.51 ± 0.015 d−	0.55 ± 0.011 d+
2 September	0.54 ± 0.005 a+	0.54 ± 0.013 a+	0.61 ± 0.018 b+	0.57 ± 0.005 b−	0.59 ± 0.008 c+	0.55 ± 0.014 c−	0.57 ± 0.013 d+	0.53 ± 0.008 d−

Note: One-way ANOVA was used to compare the differences between mean values (*p* < 0.05); the table shows the results within the same asexual lineage. a+/a− indicates that there was a significant difference in LK29 between low altitude and high altitude; b+/b− indicates that there was a significant difference in LK15 between low altitude and high altitude; c+/c− indicates that there was a significant difference in KH13 between low altitude and high altitude; and d+/d− indicates that there was significant difference in KH03 between low altitude and high altitude.

**Table 3:** Effect of altitude on the fatty acid composition of mature *C. chekiangoleosa* seed oil (relative percentage, %).

	LK29		LK15		KH13		KH03	
	High Altitude	Low Altitude	High Altitude	Low Altitude	High Altitude	Low Altitude	High Altitude	Low Altitude
12:0	0.96 ± 0.16 a+	0.05 ± 0.008 a−	0.04 ± 0.003 b−	0.24 ± 0.01 b+	0.28 ± 0.02 c+	0.02 ± 0.003 c−	0.26 ± 0.02 d+	0.1 ± 0.004 d−
14:0	0.11 ± 0.02 a+	0.08 ± 0.009 a−	0.12 ± 0.01 b−	0.15 ± 0.01 b+	0.09 ± 0.002	0.11 ± 0.003	0.09 ± 0.01	0.11 ± 0.01
16:0	8.7 ± 0.29 a−	10.27 ± 0.51 a+	9.3 ± 0.28	9.72 ± 0.22	10.76 ± 0.34 c+	9.49 ± 0.43 c−	8.76 ± 0.34	9.52 ± 0.51
9 t16:1	0.04 ± 0.002	0.04 ± 0.002	0.05 ± 0.001 b+	0.03 ± 0.001 b−	0.06 ± 0.003 c+	0.05 ± 0.001 c−	0.06 ± 0.003 d+	0.03 ± 0.001 d−
9 c16:1	0.12 ± 0.01	0.11 ± 0.01	0.11 ± 0.005 b+	0.08 ± 0.001 b−	0.09 ± 0.001 c+	0.07 ± 0.001 c−	0.12 ± 0.01 d+	0.08 ± 0.001 d−
17:0	0.11 ± 0.01	0.11 ± 0.008	0.08 ± 0.004	0.09 ± 0.003	0.09 ± 0.006 c−	0.12 ± 0.01 c+	0.08 ± 0.01 d−	0.11 ± 0.01 d+
10 c17:1	0.11 ± 0.002 a+	0.06 ± 0.001 a−	0.07 ± 0.003 b−	0.08 ± 0.004 b+	0.06 ± 0.004 c−	0.07 ± 0.002 c+	0.07 ± 0.003 d−	0.08 ± 0.001 d+
18:0	5.29 ± 0.26	5.74 ± 0.27	5.79 ± 0.13	6.08 ± 0.35	4.58 ± 0.21	4.59 ± 0.31	5.47 ± 0.21	5.49 ± 0.35
9 c18:1	78.93 ± 2.59	78.77 ± 1.02	77.72 ± 1.55	77.27 ± 0.7	75.74 ± 1.38	78.19 ± 0.41	77.36 ± 1.85	79.09 ± 1.01
18:2 n-6	5.2 ± 0.17 a+	4.27 ± 0.26 a−	6.22 ± 0.22	5.58 ± 0.38	7.63 ± 0.46 c+	6.71 ± 0.11 c−	7.21 ± 0.27 d+	4.86 ± 0.33 d−
18:3 n-3	0.43 ± 0.03	0.50 ± 0.02	0.49 ± 0.04	0.68 ± 0.53	0.61 ± 0.03	0.59 ± 0.02	0.52 ± 0.03	0.53 ± 0.46

Note: One-way ANOVA was used to compare the differences between mean values (*p* < 0.05); the table shows the results within the same asexual lineage. a+/a− indicates that there was a significant difference in LK29 between low altitude and high altitude; b+/b− indicates that there was a significant difference in LK15 between low altitude and high altitude; c+/c− indicates that there was a significant difference in KH13 between low altitude and high altitude; and d+/d− indicates that there was significant difference in KH03 between low altitude and high altitude.

**Table 4:** AUC values for each parameter.

Parameters	AUC
α-tocopherol	0.875 *
Squalene	0.813 *
β-sitosterol	0.844 *
β-amyrinol	0.688
Oil yield	0.938 *
Total flavonoids	0.750
Total polyphenols	0.625
16:0	0.750
18:0	0.688
9 c18:1	0.688
18:2 n-6	0.823 *
18:3 n-3	0.750

*: Critical parameters with AUC > 0.8.

## Data Availability

The original contributions presented in the study are included in the article/Supplementary Material, further inquiries can be directed to the corresponding authors.

## Supplementary Materials

The following supporting information can be downloaded at: https://www.mdpi.com/article/10.3390/foods14132266/s1, Table S1: LK29 fatty acid compositions during the ripening of *C. chekiangoleosa* (Relative percentage %); Table S2: LK15 fatty acid compositions during the ripening of *C. chekiangoleosa*(Relative percentage %); Table S3: LK13 fatty acid compositions during the ripening of *C. chekiangoleosa* (Relative percentage %); Table S4 LK03 fatty acid compositions during the ripening of *C. chekiangoleosa* (Relative percentage %).
