# Supplementary material for: Influence of Environmental Conditions Associated with Low and High Altitudes on Economic and Quality Characteristics of Fruit Ripening of Camellia chekiangoleosa Hu
Source: Foods. 2025 Jun 26;14(13):2266. doi: 10.3390/foods14132266 (PMC12249395; doi:10.3390/foods14132266)
Supplement: Supplementary file 1 [file foods-14-02266-s001.zip › foods-3661508-supplementary.pdf]

Table S1 LK29 fatty acid compositions during the ripening of *C. chekiangoleosa* (Relative percentage %)

	5 Jul		19 Jul		3 Aug		17 Aug		2 Sep	
	LK29H	LK29L	LK29H	LK29L	LK29H	LK29L	LK29H	LK29L	LK29H	LK29L
12:0	0.16±0.01	0.06±0.004	0.08±0.01	0.11±0.006	0.27±0.02	0.04±0.002	0.31±0.17	0.05±0.005	0.96±0.16	0.05±0.008
14:0	0.07±0.002	0.08±0.002	0.06±0.003	0.12±0.01	0.04±0.002	0.11±0.01	0.05±0.001	0.13±0.007	0.11±0.02	0.08±0.009
16:0	18.26±0.36	14.68±0.39	14.39±0.49	13.52±0.45	12.16±0.22	15.17±0.51	10.8±0.24	13.13±0.41	8.7±0.29	10.27±0.51
9t16:1	0.08±0.003	0.12±0.01	0.11±0.004	0.05±0.003	0.12±0.01	0.03±0.004	0.1±0.002	0.08±0.004	0.04±0.002	0.04±0.002
9c16:1	0.11±0.003	0.13±0.01	0.16±0.01	0.08±0.01	0.07±0.003	0.12±0.008	0.09±0.003	0.14±0.009	0.12±0.01	0.11±0.01
17:0	0.14±0.005	0.06±0.002	0.09±0.002	0.11±0.004	0.13±0.01	0.07±0.005	0.1±0.02	0.08±0.01	0.11±0.01	0.11±0.008
10c17:1	0.05±0.001	0.05±0.002	0.08±0.002	0.08±0.003	0.06±0.002	0.07±0.001	0.09±0.003	0.08±0.007	0.11±0.002	0.06±0.001
18:0	3.19±0.14	3.71±0.24	2.68±0.13	4.52±0.06	7.83±0.22	4.29±0.17	5.58±0.24	5.59±0.24	5.29±0.26	5.74±0.27
9c18:1	63.89±2.31	61.38±1.49	67.8±1.49	64.29±1.22	70.99±1.13	61.68±2.35	77.77±2.85	73.17±1.67	78.93±2.59	78.77±1.02
18:2n-6	12.96±0.22	17.62±0.58	13.87±0.26	15.83±0.62	7.62±0.13	17.59±0.11	4.77±0.36	7.10±0.37	5.2±0.17	4.27±0.26
18:3n-3	1.09±0.03	2.11±0.06	0.68±0.02	1.29±0.09	0.71±0.03	0.83±0.05	0.35±0.02	0.45±0.03	0.43±0.03	0.50±0.02

Table S2 LK15 fatty acid compositions during the ripening of *C. chekiangoleosa*(Relative percentage %)

	5 Jul		19 Jul		3 Aug		17 Aug		2 Sep	
	LK15H	LK15L	LK15H	LK15L	LK15H	LK15L	LK15H	LK15L	LK15H	LK15L
12:0	0.09±0.01	0.13±0.01	0.16±0.01	0.16±0.01	0.08±0.004	0.09±0.005	0.74±0.17	0.84±0.08	0.04±0.003	0.24±0.01
14:0	0.09±0.003	0.11±0.01	0.18±0.01	0.09±0.003	0.15±0.01	0.16±0.01	0.17±0.01	0.09±0.002	0.12±0.01	0.15±0.01
16:0	16.27±0.34	12.86±0.55	15.47±0.29	11.74±0.39	12.39±0.22	14.29±0.26	14.18±0.27	12.95±0.12	9.3±0.28	9.72±0.22
9t16:1	0.04±0.002	0.05±0.001	0.06±0.001	0.05±0.002	0.04±0.002	0.07±0.001	0.01±0.001	0.02±0.001	0.05±0.001	0.03±0.001
9c16:1	0.14±0.01	0.07±0.004	0.16±0.007	0.08±0.006	0.12±0.01	0.04±0.003	0.11±0.008	0.08±0.002	0.11±0.005	0.08±0.001
17:0	0.06±0.002	0.16±0.01	0.14±0.003	0.13±0.01	0.08±0.004	0.17±0.007	0.09±0.003	0.11±0.01	0.08±0.004	0.09±0.003
10c17:1	0.14±0.01	0.04±0.001	0.08±0.003	0.11±0.004	0.05±0.001	0.06±0.004	0.08±0.002	0.08±0.003	0.07±0.003	0.08±0.004
18:0	3.69±0.16	3.04±0.04	4.24±0.15	3.18±0.01	5.18±0.21	5.22±0.12	4.25±0.22	4.95±0.15	5.79±0.13	6.08±0.35
9c18:1	55.77±2.16	67.63±0.57	59.96±1.48	68.35±0.62	63.17±1.37	68.95±0.48	74.44±1.52	75.16±0.24	77.72±1.55	77.27±0.7
18:2n-6	14.72±0.47	14.85±0.17	11.29±0.26	15.17±0.26	9.61±0.31	10.28±0.38	5.38±0.39	5.31±0.43	6.22±0.22	5.58±0.38
18:3n-3	1.49±0.03	1.06±0.04	1.07±0.04	0.94±0.03	0.83±0.03	0.67±0.04	0.54±0.008	0.42±0.02	0.49±0.04	0.68±0.53

Table S3 LK13 fatty acid compositions during the ripening of *C. chekiangoleosa* (Relative percentage %)

	5 Jul		19 Jul		3 Aug		17 Aug		2 Sep	
	KH13H	KH13L	KH13H	KH13L	KH13H	KH13L	KH13H	KH13L	KH13H	KH13L
12:0	0.08±0.002	0.06±0.002	0.14±0.01	0.11±0.01	0.09±0.003	0.09±0.004	0.14±0.03	0.27±0.01	0.28±0.02	0.02±0.003
14:0	0.06±0.001	0.14±0.01	0.04±0.001	0.13±0.004	0.09±0.002	0.07±0.002	0.02±0.001	0.09±0.004	0.09±0.002	0.11±0.003
16:0	12.71±0.46	13.35±0.18	13.84±0.52	14.13±0.26	11.06±0.18	10.94±0.29	11.72±0.63	11.55±0.13	10.76±0.34	9.49±0.43
9t16:1	0.06±0.002	0.07±0.002	0.13±0.01	0.02±0.001	0.09±0.004	0.04±0.001	0.02±0.002	0.04±0.003	0.06±0.003	0.05±0.001
9c16:1	0.11±0.002	0.14±0.01	0.03±0.003	0.09±0.003	0.04±0.001	0.04±0.002	0.08±0.005	0.1±0.002	0.09±0.001	0.07±0.001
17:0	0.09±0.002	0.07±0.002	0.13±0.003	0.13±0.01	0.08±0.002	0.07±0.002	0.11±0.003	0.1±0.005	0.09±0.006	0.12±0.01
10c17:1	0.04±0.003	0.08±0.002	0.01±0.001	0.04±0.001	0.07±0.003	0.05±0.002	0.09±0.002	0.09±0.01	0.06±0.004	0.07±0.002
18:0	3.16±0.13	6.17±0.11	5.27±0.16	4.81±0.09	6.38±0.11	5.13±0.13	5.24±0.24	5.6±0.17	4.58±0.21	4.59±0.31
9c18:1	67.04±1.55	70.93±0.46	65.17±1.73	72.99±0.19	72.49±1.95	75.48±0.27	75.02±3.16	73.08±0.68	75.74±1.38	78.19±0.41
18:2n-6	15.38±0.22	8.12±0.13	14.29±0.13	6.53±0.09	8.73±0.27	7.16±0.15	7.00±0.17	8.47±0.34	7.63±0.46	6.71±0.11
18:3n-3	1.27±0.04	0.87±0.05	0.95±0.06	1.02±0.07	0.88±0.01	0.93±0.08	0.55±0.01	0.59±0.02	0.61±0.03	0.59±0.02

Table S4 LK03 fatty acid compositions during the ripening of *C. chekiangoleosa* (Relative percentage %)

	5 Jul		19 Jul		3 Aug		17 Aug		2 Sep	
	KH03H	KH03L	KH03H	KH03L	KH03H	KH03L	KH03H	KH03L	KH03H	KH03L
12:0	0.12±0.01	0.1±0.01	0.09±0.01	0.11±0.01	0.11±0.02	0.08±0.003	0.4±0.1	0.54±0.05	0.26±0.02	0.1±0.004
14:0	0.08±0.002	0.14±0.01	0.15±0.01	0.13±0.01	0.08±0.01	0.14±0.01	0.03±0.01	0.11±0.003	0.09±0.01	0.11±0.01
16:0	14.27±0.35	15.62±0.27	15.32±0.43	11.86±0.45	12.35±0.2	12.96±0.5	13.32±0.6	13.1±0.15	8.76±0.34	9.52±0.51
9t16:1	0.07±0.002	0.09±0.01	0.08±0.002	0.02±0.003	0.09±0.01	0.04±0.001	0.07±0.001	0.04±0.002	0.06±0.003	0.03±0.001
9c16:1	0.11±0.003	0.11±0.01	0.11±0.003	0.08±0.003	0.1±0.001	0.05±0.004	0.08±0.01	0.13±0.01	0.12±0.01	0.08±0.001
17:0	0.08±0.003	0.08±0.01	0.1±0.002	0.13±0.01	0.12±0.01	0.12±0.01	0.09±0.02	0.08±0.01	0.08±0.01	0.11±0.01
10c17:1	0.05±0.002	0.05±0.002	0.01±0.001	0.09±0.004	0.07±0.001	0.05±0.004	0.08±0.002	0.08±0.01	0.07±0.003	0.08±0.001
18:0	3.68±0.13	3.06±0.15	3.34±0.14	4.12±0.02	6.16±0.12	4.35±0.14	5.08±0.24	5.49±0.17	5.47±0.21	5.49±0.35
9c18:1	66.78±1.94	65.64±1.48	66.31±1.59	66.9±0.45	70.53±1.8	69.42±1.51	74.51±2.28	73.29±1.16	77.36±1.85	79.09±1.01
18:2n-6	13.61±0.47	13.12±0.36	13.53±0.25	15.45±0.32	9.57±0.28	12.1±0.17	5.95±0.32	6.63±0.36	7.21±0.27	4.86±0.33
18:3n-3	1.15±0.03	1.99±0.05	0.96±0.06	1.11±0.08	0.82±0.03	0.69±0.07	0.39±0.01	0.51±0.02	0.52±0.03	0.53±0.46
